# Mechanistic Basis and Clinical Evidence for the Applications of Nicotinamide (Niacinamide) to Control Skin Aging and Pigmentation

**DOI:** 10.3390/antiox10081315

**Published:** 2021-08-21

**Authors:** Yong Chool Boo

**Affiliations:** Department of Molecular Medicine, School of Medicine, BK21 Plus KNU Biomedical Convergence Program, Cell and Matrix Research Institute, Kyungpook National University, Daegu 41944, Korea; ycboo@knu.ac.kr; Tel.: +82-53-420-4946

**Keywords:** nicotinamide, niacinamide, vitamin B3, skin aging, pigmentation, cosmetic, cosmeceutical, metabolism, antioxidant, senescence, inflammation

## Abstract

Vitamin B3 (nicotinic acid, niacin) deficiency causes the systemic disease pellagra, which leads to dermatitis, diarrhea, dementia, and possibly death depending on its severity and duration. Vitamin B3 is used in the synthesis of the NAD^+^ family of coenzymes, contributing to cellular energy metabolism and defense systems. Although nicotinamide (niacinamide) is primarily used as a nutritional supplement for vitamin B3, its pharmaceutical and cosmeceutical uses have been extensively explored. In this review, we discuss the biological activities and cosmeceutical properties of nicotinamide in consideration of its metabolic pathways. Supplementation of nicotinamide restores cellular NAD^+^ pool and mitochondrial energetics, attenuates oxidative stress and inflammatory response, enhances extracellular matrix and skin barrier, and inhibits the pigmentation process in the skin. Topical treatment of nicotinamide, alone or in combination with other active ingredients, reduces the progression of skin aging and hyperpigmentation in clinical trials. Topically applied nicotinamide is well tolerated by the skin. Currently, there is no convincing evidence that nicotinamide has specific molecular targets for controlling skin aging and pigmentation. This substance is presumed to contribute to maintaining skin homeostasis by regulating the redox status of cells along with various metabolites produced from it. Thus, it is suggested that nicotinamide will be useful as a cosmeceutical ingredient to attenuate skin aging and hyperpigmentation, especially in the elderly or patients with reduced NAD^+^ pool in the skin due to internal or external stressors.

## 1. Introduction

The primary characteristic of the skin is that it surrounds our body and is directly exposed to the external environment. Skin serves the barrier function to protect the body from external harmful factors and to prevent water loss from the body, as well as the thermoregulation function to keep body temperature constant despite changes in external temperature [1]. However, when the skin is subjected to pathological conditions due to internal and external factors, such as malnutrition, infection, wounds, and exposure to pollutants, abnormalities in the immune system and excessive inflammatory response throughout the body can be induced. Even if the symptoms are limited to the skin, various skin diseases, aging, and cancer can occur, and these are the main research subjects in dermatology. 

Another characteristic of the skin is that it is an externally visible organ, and therefore, its health is important from an aesthetic point of view, as well as a medical point of view. The aging of the skin involves both the decline of various biological functions and changes in morphological beauty. In the cosmetic field, skin aging is being studied by dividing it into natural aging, which is a chronological skin change caused by internal factors of the body, and photoaging, which is a skin change caused by exposure to ultraviolet (UV) rays from the sun [2]. Natural aging and photoaging are not mutually exclusive but partly overlap. Clinical observations show that naturally aged skin is thin, dry, and has many fine wrinkles, whereas photoaged skin usually has a leathery and saggy appearance with reduced elasticity, uneven pigmentation, coarse and deep wrinkles, and telangiectasia (appearance of blood vessels) [3]. Changes due to natural aging or photoaging occur in both epidermal and dermal compartments. A decrease in the extracellular matrix (ECM) of the dermis, such as collagen and elastin, is consistently observed in either natural aging or photoaging [4].

Reactive oxygen species (ROS) and free radicals generated over the antioxidant capacity of cells due to external factors, such as UV radiation and pollution, or internal metabolic dysfunction can cause oxidative damage to cells [5]. ROS also induces senescence of cells and degradation of ECM involved in premature skin aging [6,7]. Indeed, ROS plays a pathological role in the development of various skin diseases and cancer [8,9,10]. Therefore, ingredients that directly remove ROS or enhance the antioxidant capacity of cells are expected to help maintain skin homeostasis [11,12].

Nicotinamide (niacinamide) is the amide form of water-soluble vitamin B3 (nicotinic acid, niacin). Vitamin B3 deficiency causes pellagra [13,14]. Nicotinamide is a component of coenzymes, such as nicotinamide adenine dinucleotide (NAD^+^), reduced nicotinamide adenine dinucleotide (NADH), nicotinamide adenine dinucleotide phosphate (NADP^+^), and reduced nicotinamide adenine dinucleotide phosphate (NADPH) [14,15]. Nicotinamide has the same vitamin activity as nicotinic acid, but other pharmacological actions and side effects are different [16]. Unlike nicotinic acid, nicotinamide does not reduce cholesterol or cause flushing [17]. Supplementation of nicotinamide as an essential nutrient will be beneficial to the health of the whole body and the skin. 

In the field of dermatology, many studies on nicotinamide and its analogs have been reported concerning the prevention and treatment of cancer, blistering disorders, acne vulgaris, psoriasis, wound healing, and pigmentation disorders [18,19,20,21]. Nicotinamide has also been used in the cosmetic field for decades to prevent skin aging and brighten skin tone [22,23,24,25]. However, its mechanism of action in alleviating skin diseases or controlling skin aging and pigmentation is not well understood. In addition, it is unclear whether the efficacy of nicotinamide is its direct effect or its indirect effect acting as a precursor of other active metabolites.

The purpose of this review is to understand the mechanistic basis for the cosmeceutical application of nicotinamide. The pharmaceutical application of nicotinamide is excluded from the scope of this review. First, we briefly review the metabolic process of nicotinamide. Secondly, we examine the antioxidant and anti-inflammatory effects of nicotinamide and its effects on cell senescence and epidermal differentiation. Next, we discuss the effects of nicotinamide on the ECM and skin barrier, which is closely related to skin aging, and on the synthesis and distribution of melanin related to skin pigmentation. Finally, we review the results of clinical trials on the efficacy of cosmetic formulations containing nicotinamide alone or in combination with other active ingredients to control skin aging and pigmentation. It is hoped that this study will help us understand the mechanism of action of nicotinamide and correctly evaluate the potential of nicotinamide as a cosmeceutical.

## 2. Metabolism of Nicotinamide

### 2.1. Synthesis and Function of NAD(H) and NADP(H)

**Figure 1:**
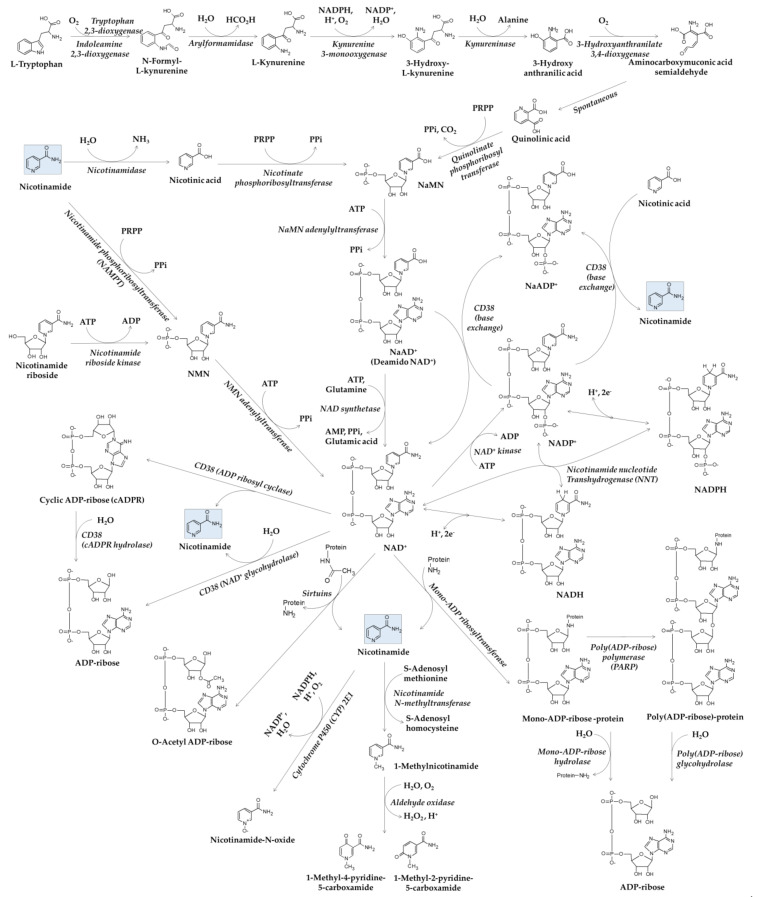
Metabolic pathways related to nicotinamide: ADP, adenosine diphosphate; AMP, adenosine monophosphate; ATP, adenosine triphosphate; cADPR, cyclic ADP-ribose; CYP, cytochrome P450; NaAD^+^, nicotinic acid adenine dinucleotide; NaADP^+^, nicotinic acid adenine dinucleotide phosphate; NAD^+^, nicotinamide adenine dinucleotide; NADH, reduced nicotinamide adenine dinucleotide; NADP^+^, nicotinamide adenine dinucleotide phosphate; NADPH, reduced nicotinamide adenine dinucleotide phosphate; NaMN, nicotinic acid mononucleotide; NAMPT, nicotinamide phosphoribosyltransferase; NMN, nicotinamide mononucleotide; NNT, nicotinamide nucleotide transhydrogenase; PARP, poly(ADP-ribose) polymerase; PPi, inorganic pyrophosphate; PRPP, phosphoribosyl pyrophosphate.

The roles of NAD^+^ coenzyme in the life system are significant and broad [15,26]. In this section, we briefly review the metabolic pathways of NAD^+^ with special attention to nicotinamide (Figure 1). 

De novo synthesis of NAD^+^ starts with oxidation of L-tryptophan to N-formyl-L-kynurenine by tryptophan 2,3-dioxygenase or indoleamine 2,3-dioxygenase [27,28]. N-formyl-L-kynurenine is converted to quinolinic acid via multiple enzymatic and non-enzymatic reactions. Nicotinic acid mononucleotide (NaMN) is synthesized from quinolinic acid and phosphoribosyl pyrophosphate (PRPP) by quinolinate phosphoribosyltransferase, and inorganic pyrophosphate (PPi) and carbon dioxide are released as by-products. 

As tryptophan is one of the essential amino acids that cannot be synthesized well by humans, the salvage pathway using nicotinamide or nicotinic acid from dietary sources is important for the synthesis of NAD^+^ [29]. NaMN is synthesized from nicotinic acid and PRPP by nicotinate phosphoribosyltransferase, and PPi is released as a by-product. Nicotinic acid adenine dinucleotide (NaAD^+^) is synthesized from NaMN and adenosine triphosphate (ATP) by NaMN adenylyltransferase, releasing PPi as a by-product and is then used in the synthesis of NAD^+^ by NAD synthetase, which uses glutamine as an amine group donor and energy from ATP hydrolysis to adenosine monophosphate (AMP) and PPi. NAD^+^ is also synthesized by NMN adenylyltransferase using nicotinamide mononucleotide (NMN) and ATP. Nicotinamide and PRPP are used in the synthesis of NMN by nicotinamide phosphoribosyltransferase (NAMPT), which releases PPi as a by-product. NMN is also synthesized from nicotinamide riboside and ATP by nicotinamide riboside kinase, which releases adenosine diphosphate (ADP) as a by-product. 

Conversion of NAD^+^ to NADP^+^ is catalyzed by NAD^+^ kinase consuming ATP molecules for needed free energy [30]. Nicotinamide nucleotide transhydrogenase (NNT) catalyzes a reversible reaction, NADH + NADP^+^ ⇌ NAD^+^ + NADPH [31]. NAD(H) and NADP(H) play as cofactors or coenzymes in a myriad of oxidation-reduction reactions in biological systems [32]. NAD^+^ serves as an electron acceptor in many enzyme reactions in glycolysis, the citric acid cycle, and β-oxidation of fatty acids, producing NADH. NADH serves as an electron donor in many enzyme reactions, such as NADH dehydrogenase in complex Ι of mitochondrial electron transport and lactate dehydrogenase in the cytosol. NADP^+^ serves as an electron acceptor in many enzyme reactions, such as glucose 6-phosphate dehydrogenase in the pentose phosphate pathway and isocitrate dehydrogenase outside the context of the citric acid cycle. NADPH serves as an electron donor in many enzyme reactions, such as NADPH oxidase, cytochrome P450 (CYP), nitric oxide synthase, and glutathione reductase. Glutathione reductase catalyzes a reaction, glutathione disulfide (GSSG) + NADPH → 2 × glutathione (GSH) + NADP^+^.

### 2.2. Metabolisms of NAD^+^ and Nicotinamide

Poly(ADP-ribose) polymerase (PARP) family consists of 18 genes, which encode 17 enzymes with either mono-ADP ribosyltransferase or PARP activity [33]. NAD^+^ is used as a substrate for mono-ADP-ribosylation of target proteins catalyzed by mono-ADP ribosyltransferase activity, and for poly(ADP-ribose) polymerization catalyzed by PARP activity [34,35]. Nicotinamide is released as a by-product. Hydrolysis of mono- and poly-ADP-ribosylated proteins by mono-ADP-ribose hydrolase and poly(ADP-ribose) hydrolase results in the production of ADP-ribose. These reversible processes are involved in DNA repair, apoptosis, and many other biological processes to maintain cellular homeostasis [36].

Sirtuins are a family of signaling proteins that have a mono-ADP-ribosyltransferase activity or a protein deacylase activity (deacetylase, desuccinylase, demalonylase, demyristoylase, or depalmitoylase activity) and have been hypothesized to play a role in the aging process [37]. Histone deacetylation by sirtuins yields the deacetylated protein, O-acetyl ADP-ribose, and nicotinamide [38]. Sirtuins epigenetically regulate target gene expression involved in stress resistance and energy alertness and directly link cell physiology to the energy status of the cell [39]. 

CD38 functions as a receptor and a multifunctional enzyme, catalyzing the cleavage of NAD^+^ into cyclic ADP ribose (cADPR) and nicotinamide, the hydrolysis of cADPR to ADP-ribose, and the direct hydrolysis of NAD^+^ to ADP-ribose and nicotinamide [40]. CD38 also catalyzes a base exchange reaction that couples the conversion of NADP^+^ to NaADP with the conversion of nicotinic acid to nicotinamide [41], or that of NaAD^+^ to NAD^+^ [42]. Both cADPR and NaADP^+^ play essential roles for the regulation of intracellular Ca^2+^ [43,44]. CD38 is considered to play a critical role in keeping a harmonized balance between various NAD^+^ metabolites. 

Nicotinamide is metabolized to 1-methylnicotinamide by nicotinamide N-methyltransferase, which uses S-adenosyl methionine as a methyl group donor [45]. 1-Methylnicotinamide is further oxidized to 1-methyl-2-pyridone-5-carboxamide or 1-methyl-4-pyridone-3-carboxamide by aldehyde oxidase [46]. Nicotinamide is also directly oxidized to nicotinamide N-oxide by CYP 2E1 in human liver microsomes [47]. These enzyme reactions mainly occur in the liver and are considered a clearance mechanism involved in the urinary excretion of nicotinamide. 

## 3. Antioxidant and Anti-Inflammatory Effects of Nicotinamide

### 3.1. Antioxidant Properties of Nicotinamide

Lngestion of nicotinamide, prevents lipid peroxidation and normalizes the reduced antioxidants and antioxidant enzymes in experimental animal models [48,49,50]. 

Kamat et al. showed that nicotinamide scavenged singlet oxygen at the rate constant of 1.8 × 10^8^ M^−1^ s^−1^ and inhibited lipid peroxidation of rat liver microsomes induced by the photosensitized reaction of methylene blue irradiated with visible light in the presence of oxygen [51]. They also showed that nicotinamide inhibited lipid peroxidation induced by NADPH/ADP-Fe^3+^ in rat liver microsomes [51]. Nicotinamide inhibited lipid peroxidation and protein oxidation (carbonylation) induced by the ascorbate–Fe^2+^ system in the rat brain mitochondria, whereas such action was not observed for nicotinic acid [52]. 

### 3.2. Protective Effect of Nicotinamide in Cells Exposed to Environmental Stressors

Nicotinamide rescued the viability of a Chinese hamster ovary cell line (CHO AA8) irradiated with UV radiation and prevented apoptosis through mechanisms related to the stabilization of the cytoskeleton proteins, such as F-actin, vimentin, and β-tubulin [53]. Nicotinamide exhibited a protective effect against UVA- and/or UVB-induced DNA damage in normal human epidermal melanocytes, as indicated by decreased levels of cyclobutane pyrimidine dimers and 8-hydroxy-2’-deoxyguanosine [54]. This effect was associated with the enhanced expression of nucleotide excision repair genes, such as sirtuin 1 (SIRT1), tumor suppressor protein P53, damage-specific DNA binding protein (DDB) 1 and 2, 8-oxoguanine glycosylase (OGG) 1, excision repair cross-complementation group (ERCC) 1 and 2, and cyclin-dependent kinase (CDK) 7, and the activation of the nuclear factor erythroid 2-related factor 2 (NRF 2) signaling pathway.

Nicotinamide inhibited the generation of ROS, the oxidation of lipids, proteins, and DNA, cell membrane depolarization, and the apoptosis in human HaCaT keratinocytes cells exposed to particulate matter (PM) 2.5 [55]. Mi et al. examined the protective effect of nicotinamide and 12-hydroxystearic acid in reconstructed human skin equivalents exposed to benzo(a)pyrene as a representative airborne particle-bound organic compound, or to squalene monohydroperoxide as a representative sebum peroxidation product [56]. Individual treatment and co-treatment of the skin equivalents with nicotinamide (5 mM) and 12-hydroxystearic acid (20 μM) ameliorated viability loss, inflammatory response, and pigmentation induced by benzo(a)pyrene or squalene monohydroperoxide. 

These studies suggest that the topical application of nicotinamide may alleviate oxidative stress and reduce cytotoxicity, inflammation, and pigmentation in the skin that is exposed to UV or PM.

### 3.3. Anti-Inflammatory Effects of Nicotinamide

Nicotinamide suppressed interleukin (IL)-8 production at the mRNA and protein levels through modulation of the nuclear factor (NF)-κB and mitogen-activated protein kinase (MAPK) pathways in HaCaT cells and primary keratinocytes stimulated by *Propionibacterium acnes*, the etiological agent causing inflammatory acne vulgaris [57]. Nicotinamide downregulated the expression of IL-6, IL-10, monocyte chemoattractant protein-1 and tumor necrosis factor (TNF)-α in UV-irradiated keratinocytes [58]. 

Nicotinamide attenuated the synthesis of inflammatory mediators, such as prostaglandin (PG) E_2_, IL-6, and IL-8 in human epidermal keratinocytes and in full-thickness three-dimensional skin organotypic models that were stimulated by UV radiation [59]. In a clinical trial, pretreatment with 5% nicotinamide reduced erythema that was induced by UV radiation [59]. Analysis of IL-1α and its receptor antagonist (IL-1αRA) ratios showed that nicotinamide significantly reduced the UV-induced inflammatory response, compared to the control sites.

### 3.4. Anti-Inflammatory Effects of N-Methylnicotinamide and NMN

Nicotinamide and l-methylnicotinamide exhibit anti-inflammatory effects in several experimental models although the relative activity of these two substances is not consistent. The contact hypersensitivity reaction of CBA/J inbred mice to oxazolone was reduced when the mice were fed with 1-methylnicotinamide or nicotinamide added to the drink, the former being relatively more effective [60]. In an in vitro experiment using CBA/J mouse peritoneal macrophages activated with lipopolysaccharide, nicotinamide inhibited the production of a variety of pro-inflammatory factors, such as TNF-α, IL-6, nitric oxide, and PGE_2_, and 1-methylnicotinamide was less effective, although both substances similarly attenuated the generation of ROS [61]. It is considered that the anti-inflammatory activity of these two substances is affected by bioavailability, such as absorption through the digestive tract and cell membrane. 

In clinical trials, topical application of a gel containing 0.25% 1-methylnicotinamide twice a day for 4 weeks alleviated rosacea, a chronic facial dermatosis [62]. Intradermal injection of 1-methylnicotinamide or nicotinamide increased skin vascular permeability in rats, the former being more effective [63]. The changes in skin vascular permeability were attenuated by indomethacin and N_ω_-nitro-L-arginine methyl ester, indicating the involvement of PGs and nitric oxide (NO). Although the molecular mechanism linking skin vascular permeability and rosacea is unclear, it is considered that 1-methylnicotinamide or nicotinamide directly or indirectly affects vascular endothelial function [64,65].

In a rat model, oral administration of NMN alone or in combination with *Lactobacillus fermentum* TKSN041 reduced UV-induced skin oxidative damage and inflammatory response and restored small molecular antioxidants and antioxidant enzymes in blood and skin tissues [66].

## 4. Modulation of Cell Senescence and Epidermal Differentiation by Nicotinamide 

### 4.1. Differential Effects of Nicotinamide on Lifespans of Yeast and Mammalian Cells

Nicotinamide is known as an inhibitor of silent information regulator-2 (sir2) deacetylase that mediates lifespan extension by calorie restriction in yeasts (*Saccharomyces cerevisiae*), and nicotinamide depletion or overexpression of nicotinamidase 1 (pyrazinamidase 1) prolongs the lifespan of yeast cells [67]. 

On the contrary, nicotinamide supplementation to human cells rather prolongs the replicative lifespan and retards the senescence [68,69]. Matuoka et al. observed that nicotinamide reverses the aging phenotypes in human diploid fibroblasts as evaluated by cell morphology, senescence-associated β-galactosidase activity, and cell replication potential, and tentatively attributed this action of nicotinamide to the enhancement of histone acetyltransferase activity and subsequently altered gene expression [68]. Lim et al. demonstrated that that nicotinamide extends the lifespan of primary human diploid somatic fibroblasts (82-6 and IMR-90) via a mechanism largely independent of SIRT1, a close human homolog of yeast sir2 [69]. 

The discrepancy regarding the effects of nicotinamide on the lifespans of yeast cells vs. mammalian cells could be attributed to differences in intracellular nicotinamide concentrations in situ [70,71]. The 50% inhibitory concentration of nicotinamide on Sir2 is about 50 μM, and this level of nicotinamide concentration can be reached in yeasts [70]. On the other hand, it is difficult to reach this nicotinamide concentration in mammalian cells because the supplied nicotinamide is rapidly metabolized by NAMPT in a mammalian NAD^+^ salvage pathway [71]. 

### 4.2. Antisenescence Effects of Nicotinamide 

Cellular NAD^+^ pool is low in aged skin [72]. Thus, external supplementation of nicotinamide as a primary precursor of NAD^+^ and related coenzymes may improve the epidermal homeostasis and cellular bioenergetics in aged and stressed cells [73]. Kang et al. proposed that the extension of the lifespan of normal human fibroblasts by nicotinamide might be associated with the reduction in mitochondrial ROS production [74]. The antioxidant activity of nicotinamide reducing ROS production and lipofuscin accumulation correlates with antisenescence activity suppressing the increases in cell size, granule content, and senescence-associated β-galactosidase activity as observed in both rapidly senescing cells (human breast cancer MCF-7 cell line treated with Adriamycin) and already senescent cells (old passage human fibroblasts) [75]. Gene expression of subunits of complexes I to V of mitochondrial electron transport chain was reduced in fibroblasts from older aged donors, and treatment of the cells with nicotinamide restored gene expression and mitochondrial function to younger cell levels [76]. 

Ectopic expression of NAMPT in human aortic endothelial cells extended replicative lifespan, delayed markers of senescence, and limited ROS accumulation under high glucose conditions [77]. Nicotinamide protected glycolysis and oxidative phosphorylation activities in dermal fibroblasts exposed to oxidative stress through a mechanism partially dependent on NAMPT [78]. NAMPT and NAD^+^ contents have been shown to decline in primary mouse embryonic fibroblast cells undergoing replicative senescence, whereas constitutive over-expression of NAMPT increases NAD^+^ content and delays cell senescence, which is associated with increases in the activity of SIRT1 and the expression levels of superoxide dismutase 2 and catalase [79]. FK866, a NAMPT inhibitor, induced premature differentiation and senescence of human primary keratinocytes in multi-dimensional culture, and this effect was competitively attenuated by nicotinamide [80]. Therefore, NAMPT is considered to mediate the antisenescence effects of nicotinamide at least partly. 

### 4.3. Epidermal Stem Cells

Adult stem cells are present in the bulge region of the hair follicles and the basal layer of the interfollicular epidermis and play a critical role in maintaining the structural and functional integrity of the skin through self-renewal and generation of daughter cells that undergo terminal differentiation [81]. Skin aging is more associated with the reduction of healthy stem cells able to respond to proliferative signals rather than the reduction of the total number of stem cells [82]. Liu et al. have proposed a mechanistic model for skin aging based on the competition between epidermal stem cells expressing different levels of hemidesmosome component collagen 17A1 [83]. In this model, the stressed stem cells expressing a low level of collagen 17A1 are delaminated from the basal epidermis, whereas healthy stem cells expressing a high level of collagen 17A1 survive in the aging skin. Regardless of who the final winner is, this competition leads to an eventual loss of collagen 17A1 due to exhaustion of epidermal stem cells and results in skin aging represented by a thin epidermal structure. 

Epidermal stem cells can also undergo senescence, accelerating premature aging of the skin [84]. It is hypothesized that the skin aging process may be slowed down by maintaining a young stem cell phenotype [85]. In this regard, sirtuins are viewed as a promising target in slowing down the aging process [86]. Various natural compounds are known to modulate the activity of sirtuins and will be potentially useful for this purpose [87]. 

### 4.4. Modulation of Epidermal Differentiation by Nicotinamide

Nicotinamide affects the proliferation and differentiation of various stem cells including human embryonic stem cells [88]. In a study by Tan et al. [80], high concentrations of nicotinamide inhibited the differentiation of the upper epidermal layers and maintained proliferation in the basal layer of a three-dimensional organotypic skin model. Nicotinamide increased the proliferative capacity of human primary keratinocytes and the proportion of human primary keratinocyte stem cells (holoclones), which were reduced by FK866. By contrast, FK866 induced the premature senescence of human primary keratinocyte, which was rescued by nicotinamide. These observations suggest that nicotinamide metabolism through NAMPT can modulate epidermal differentiation and stem cell biology.

## 5. Enhancement of ECM and Skin Barrier by Nicotinamide

### 5.1. Changes of ECM by Skin Aging

The main component of ECM is collagen and elastin, and these fibrous components provide the skin’s tensile strength, elasticity, and resiliency [89]. In human skin, type I, type III, and type V collagen constitute 80–90%, 8–12%, and less than 5% of total collagen, respectively [90]. Alteration in the amount and structures of collagen and elastin is a common feature of natural aging and photoaging of the skin [4]. The activities of matrix metalloproteinase (MMP) and elastase are increased in aged skin while transforming growth factor (TGF)-β signaling that leads to the synthesis of collagen is reduced [91,92].

ROS increased by internal and external factors stimulates activator protein (AP)-1 and NF-κB through cell signaling systems involving several MAPKs, and increases the expression of MMPs in epidermal keratinocytes and dermal fibroblasts, resulting in collagen degradation [7,89,92,93]. Stratifin, also called 14-3-3σ protein, plays an important role in communication between keratinocytes and fibroblasts, especially for the degradation of dermal collagen associated with premature skin aging [93,94]. Stratifin from the keratinocytes exposed to UV radiation stimulates the fibroblasts in close vicinity to increase MMP 1 expression [95,96].

Other components of the ECM include different types of glycosaminoglycans, such as heparan sulfate/heparin, chondroitin sulfate/dermatan sulfate, keratan sulfate, and hyaluronan and proteoglycans made of glycosaminoglycans (other than hyaluronan) and protein cores [97]. These extremely hydrophilic components reside in the space between the cell and the collagen/elastin fibrils in the dermis and adopt highly extended conformations that enable matrices to hold a high amount of water and to withstand high compressive forces dermis [98]. The levels of different types of glycosaminoglycans and proteoglycans change differently by natural aging and photoaging of the skin [99]. 

### 5.2. Effects of Nicotinamide on Collagen and Other ECMs 

Nicotinamide and its derivatives have been shown to increase the expression of collagen (type I, III, and V), elastin, and fibrillin (1 and 2), and reduce MMP (1, 3, and 9) and elastase activity in non-irradiated and UVA-irradiated dermal fibroblasts [100,101]. Nicotinamide alone or in combination with other substances, such as L-carnosine, hesperidin, enhanced fibroblast collagen synthesis and cellular proliferation, thereby augmenting wound healing in vitro [102]. Topical nicotinamide improved tissue regeneration by increasing fibroblast proliferation, collagen synthesis, and vascularization in skin wounds of Sprague Dawley rats [103]. These studies provide evidence from various aspects that nicotinamide has the action of promoting the synthesis of dermal collagen and inhibiting its degradation.

### 5.3. Enhancement of Skin Barrier by Nicotinamide

During aging, the structural and functional integrity of the skin barrier is changed or disturbed [104]. Tanno et al. showed that in cultured human epidermal keratinocytes, nicotinamide could upregulate the synthesis of major components of skin barriers, such as ceramide, other sphingolipid fractions (glucosylceramide and sphingomyelin), free fatty acid, and cholesterol [105]. Supplementation of nicotinamide to cultured normal human epidermal keratinocytes increased the synthesis of involucrin and filaggrin, which are essential proteins for fully integral keratinized corneocytes [106]. 

A facial moisturizer containing 2% nicotinamide improved skin barriers in patients with rosacea [107]. Myristyl nicotinate enhanced the NAD^+^ pool, epidermal differentiation, and barrier function in the photoaged skin [108]. Thus, nicotinamide and its metabolism could enhance the structural and functional integrity of the skin barriers. 

## 6. Regulation of Pigmentary Process by Nicotinamide

### 6.1. Effects of Nicotinamide on Melanogenesis vs. Melanosome Transfer

Nicotinamide has a variable effect on melanin synthesis in melanocyte monoculture. There are reports that nicotinamide increased, decreased, or had no significant effect on tyrosinase activity and melanin synthesis [109,110]. On the other hand, in melanocyte-keratinocyte co-culture, reconstituted skin tissue model, or live skin, nicotinamide consistently decreased melanin content or pigmentation [22,111]. Furthermore, nicotinamide slowed the melanosome transfer from melanocytes to keratinocytes [22,111]. This suggests that the interaction between melanocytes and keratinocytes is important in skin pigmentation and that nicotinamide may affect melanocytes indirectly by primarily affecting keratinocytes.

### 6.2. Mechanisms for Melanosome Transfer

The transfer of melanosomes from melanocytes to keratinocytes has become a very important and interesting research topic in dermatology [112,113,114,115]. Melanosome transfer, the process of transferring a package of organelles from a donor cell to a recipient cell, is a very unique biological process, and various mechanisms, such as cytophagocytosis, membrane fusion, shedding–phagocytosis, and exocytosis–endocytosis, have been proposed to describe this process [115]. The membrane fusion mechanism was supported by Scott et al. [116]. Shedding–phagocytosis mechanisms were supported by Ando et al. [117] and Wu et al. [118]. The exocytosis–endocytosis mechanism was supported by Tarafder et al. [119]. Regardless of the mechanism, the precise nature of the “donate-it” signal that keratinocytes send to melanocytes and the “receive-it” signal that melanocytes send to keratinocytes is not yet clear. 

### 6.3. Modulation of Melanosome Transfer

Protease-activated receptor-2 (PAR-2) is expressed in the skin and plays an important role in the regulation of growth and differentiation of keratinocytes [120,121]. In 2000, Seiberg et al. showed that PAR-2 expressed in keratinocytes could regulate skin pigmentation, although this receptor is not expressed in melanocytes [122]. They further showed that activation of PAR-2 by SLIGRL peptide (a peptide agonist derived from the N-terminus of PAR-2) could enhance melanosome transfer and inhibition of this receptor by RWJ-503530 (a serine protease inhibitor) could reduce melanosome transfer [123].

UV rays increased PAR-2 expression in the upper epidermis of human skin, and the change was more rapid and bigger in dark-skinned people [124]. Optimized concentration of hydrogen peroxide (0.3 mM) increased melanin content and melanosome transfer in melanocyte–keratinocyte co-cultures through upregulating expression levels of PAR-2 and Rab-27A [125]. Activation of keratinocyte PAR-2 stimulated the release of PGE_2_ and PGF_2α_, the paracrine factors act on EP1, EP3, and FP receptors on melanocytes, increasing the number and length of melanocyte dendrites [126]. PGE_2_, as well as α-melanocyte-stimulating hormone (MSH), stimulated melanosome transfer in melanocyte–keratinocyte co-cultures [127]. These studies suggest a possible relationship between oxidative stress and PAR-2 activation, and thus, there is a possibility that melanosome transfer may be modulated by certain antioxidants or anti-inflammatory agents. Interestingly, macelignan, a natural product derived from *Myristica* *fragrans*, attenuated the expression of PAR-2 at the mRNA and protein levels, calcium mobilization, and phagocytic activity of HaCaT keratinocyte cells stimulated with SLIGRL peptide [128]. Macelignan also reduced PGE₂ secretion from HaCaT keratinocytes and dendrite formation of B16F10 melanoma cells in a co-culture model with SLIGRL stimulation [128]. However, it is not known whether nicotinamide affects the expression of PAR-2 and related signaling pathways.

Melanosome transfer is also regulated by other receptors, such as keratinocyte growth factor receptors expressed in keratinocytes [114], N-methyl-D-aspartate receptors [129], transient receptor potential cation channel subfamily M member 1 (TRPM1, melastatin-1) [130], and Toll-like receptors 2/3 [131] expressed in melanocytes. The direct and indirect effects of nicotinamide on cellular signaling related to melanosome transfer involving these receptors remain to be explored [132].

## 7. Clinical Evidence for Skin Antiaging Efficacy of Nicotinamide 

Clinical studies on the skin antiaging efficacy of nicotinamide alone or in combination with other active ingredients are summarized in Table 1. In double-blind, placebo-controlled, split-face, left–right, randomized clinical studies, Bissett et al. assessed the effect of nicotinamide on the appearance of aging facial skin [23,24]. Moisturizer product with or without containing 5% nicotinamide was applied on the facial skin for 12 weeks. Nicotinamide at 5% was evaluated to be well tolerated by the skin and to improve a broad array of skin appearance (fine lines/wrinkles, texture, hyperpigmentation spots, red blotchiness, and skin sallowness), and elasticity.

In a randomized, double-blind, placebo-controlled, split-face comparative study by Chiu et al. [133], 4% nicotinamide alone reduced pores and skin unevenness after 8 weeks and improved wrinkles after 12 weeks. In contrast, the formulation containing 0.03% kinetin + 4% nicotinamide significantly reduced erythema and hyperpigmented spots in addition to pores, roughness, and wrinkles after 8 weeks.

The anti-wrinkle effect of nicotinamide was further examined by Kawada et al. in a randomized, placebo-controlled, split-face study [134]. A cosmetic containing 4% nicotinamide was applied on the wrinkles of one side and a control cosmetic on the other side of the face for 8 weeks. The wrinkle grades in the test area, evaluated by doctors’ visual observation, were significantly lower than those before application (*p* < 0.001) or in the control area (*p* < 0.001) at the endpoints. The average surface roughness (Ra value) on the test area, determined using skin replica, was significantly lower, compared to the pre-application values (*p* < 0.01) or those in the control site (*p* < 0.05). 

Fu et al. reported significant results in a clinical trial comparing the antiaging effect of the cosmetic regimen using a series of products containing nicotinamide/other active ingredients plus a sunscreen with the sun protection factor (SPF) 30, vs. the use of control products containing 0.02% Tretinoin plus a sunscreen with SPF 30 [135]. However, it is difficult to estimate the relative contribution of nicotinamide to the observed antiaging effects of the cosmetic regimen.

Several other clinical trials have evaluated the efficacy of cosmetics containing nicotinamide and several other active ingredients (silk sericin, diacylglycerol, fatty alcohols, retinol, resveratrol, hexylresorcinol, and/or stem cell culture medium) [136,137,138]. These products showed a wrinkle improvement effect in common, and certain products were evaluated to have improvement effects on skin moisture, skin barrier, elasticity, surface morphology, skin clarity, and/or pigmentation [136,137,138]. Again, it is difficult to estimate the contribution of nicotinamide to the clinical trial results obtained using the combination formulation.

## 8. Clinical Evidence for Skin-Lightening Efficacy of Nicotinamide

### 8.1. Skin-Lightening Efficacy of Nicotinamide

Clinical studies on the skin-lightening efficacy of nicotinamide as an active ingredient are summarized in Table 2. Hakozaki et al. examined the skin depigmenting efficacy of nicotinamide in humans [22]. In a randomized, split-face, double-blind, paired clinical study involving eighteen Japanese women with multiple types of brown hyperpigmentation, subjects applied a test moisturizer containing 5% nicotinamide and the control moisturizer without nicotinamide to each side of the face twice daily for 8 weeks. The side of the face receiving the test moisturizer showed a significant decrease in the total hyperpigmented area measured by image analysis and a reduction in visually assessed hyperpigmentation degree, compared to the side receiving the control moisturizer after 4 weeks or 8 weeks of treatment. 

Nicotinamide has been incorporated into a sunscreen product for added performance. In total, 120 Japanese women with moderate-to-deep facial tan were enrolled in another randomized, split-face, double-blind, round-robin design and were assigned to apply any two of three different products, each product to each side of their face twice daily for eight weeks [22]. Three tested products are a vehicle moisturizer, a control sunscreen moisturizer with SPF 15, and a test sunscreen moisturizer containing 2% nicotinamide. The skin color is expressed with the Commission Internationale de l’Eclairage Lab color space composed of L* value (lightness), a* value (redness), and b* value (blueness) [141]. After 4 weeks of treatment, L* values of the treated sides were the highest for a test sunscreen moisturizer, followed by a control sunscreen moisturizer and a vehicle moisturizer, with statistical significances between test groups. After 4 weeks of treatment, the side of the face receiving a test sunscreen moisturizer showed a significant increase in visually assessed skin lightness, compared to the side receiving a vehicle moisturizer, whereas the side of the face receiving a control sunscreen moisturizer was not lighter than the vehicle moisturizer-treated side. 

Dose-dependent efficacy of nicotinamide was examined in a double-blinded, randomized, vehicle-controlled, split-face design human clinical trial that involved 79 Japanese women with multiple types of brown hyperpigmentation on both sides of the face [111]. Group 1 subjects applied a 5% nicotinamide-containing moisturizer and the vehicle moisturizer, and group 2 subjects applied a 2% nicotinamide-containing moisturizer and the vehicle moisturizer to the assigned sides of their faces twice a day for 8 weeks. After 4 or 8 weeks of treatment, the side of the face receiving a 5% nicotinamide-containing moisturizer demonstrated a higher hyperpigmented spot-reduction than the site receiving the vehicle moisturizer, while a 2% nicotinamide-containing moisturizer efficacy did not show a statistically significant effect, compared to the vehicle moisturizer. During the regression period, the spot-reduction efficacy of a 5% nicotinamide-containing moisturizer was gradually reduced to a level not statistically different from the vehicle moisturizer after 42 weeks. Therefore, topical nicotinamide can have dose-dependent and reversible skin depigmenting effects in humans. 

Depigmenting efficacy of nicotinamide was compared with other active ingredients. Navarrete-Solís et al. performed a double-blind, randomized clinical trial to compare the efficacy and safety of 4% nicotinamide vs. 4% hydroquinone in the treatment of melisma [139]. For the study, 27 melasma patients applied a product on the left side of the face, and the other on the right side for 8 weeks. After 8 weeks of treatment, melasma area and severity index (MASI) was decreased, L* value was increased and a* value was unchanged by both treatments compared to the baseline values. Good-to-excellent improvement was observed in 44% of patients receiving nicotinamide and 55% of patients receiving hydroquinone. Side effects were noted in 18% of patients receiving nicotinamide and 29% of patients receiving hydroquinone. Therefore, nicotinamide and hydroquinone were considered to have comparable skin depigmenting activity. 

Castanedo-Cazares et al. compared the efficacy of nicotinamide and desonide against axillary hyperpigmentation, which is a variant of inflammatory hyperpigmentation [140]. In a clinical trial involving 24 women with hyperpigmented axillae, the emulsions containing 4% nicotinamide or 0.05% desonide (a low potency corticosteroid used against skin inflammation) were applied to the hyperpigmented axillary region for 9 weeks. Both nicotinamide and desonide improved skin lightness, compared with placebo, and the former was slightly less effective than the latter. 

### 8.2. Skin-Lightening Efficacy of Combined Formulations of Nicotinamide and Other Active Ingredients 

An improved whitening efficacy can be expected when nicotinamide is used in combination with other active ingredients than when used alone. In addition, it will be possible to enhance the whitening efficacy by increasing the absorption of active ingredients into the skin in a special way rather than simply applying the formulation to the skin. Table 3 introduces several clinical studies that have been conducted based on this reasoning. The composite formulations additionally contain various active ingredients such as arbutin, kojic acid, ascorbic acid, ascorbyl glucoside, tranexamic acid, N-undecylenoyl phenylalanine, N-acetyl glucosamine, *trans*-4-(amino methyl) cyclohexanecarboxylic acid, potassium azeloyl diglycinate, hydroxyethylpiperazineethane sulfonic acid, and/or epidermal growth factor.

In the study of Bissett et al. [143], the whitening efficacies of 5% nicotinamide alone and combined formulation of 5% nicotinamide plus 1% N-undecylenoyl phenylalanine were compared. Significant differences were found after 8 weeks of using these products, the latter being significantly more efficacious. In several other clinical studies, the combined formulation was compared with the vehicle formulation; thus, it is difficult to distinguish the contribution of individual components. In addition, in some studies, the content of active ingredients is not disclosed, and therefore, caution is needed in interpreting the results.

Significantly superior results were observed when the composite formulation (A gel containing 2% ascorbyl glucoside, and 3.5% nicotinamide) was applied with ultrasonic treatment, compared to either conventional application only or ultrasonic treatment only [142]. This study suggests that active ingredients, as well as techniques to increase skin absorption, are important for better clinical benefit.

## 9. Discussion

### 9.1. Cosmetic Benefits and Side Effects of Topical Nicotinamide

In cosmetics, nicotinamide is mainly formulated at a concentration of 4 to 5% and is used to control skin aging and pigmentation. Several clinical trials have shown that the formulation containing nicotinamide has the effect of relieving skin aging such as wrinkles, elasticity, and skin color, compared to the control formulation that does not contain nicotine [23,134,135]. When nicotinamide-containing cosmetics are used in a well-planned regimen, you can expect a skin-aging-relieving effect comparable to that of retinol products [135]. To be sure, you can expect a good effect with a combination of nicotinamide and retinol [137]. When products containing nicotinamide are applied to hyperpigmented areas, skin-lightening effects can be expected [22,111]. The facial skin-lightening efficacy of 4% nicotinamide is almost comparable to that of 4% hydroquinone [139]. The underarm skin-lightening efficacy of 4% nicotinamide is slightly weaker than 0.5% desonide [140]. 

Nicotinamide-containing products can be used together with sunscreen products to attenuate sun-induced skin aging and pigmentation [135,143]. The skin-lightening efficacy of nicotinamide can be expected to increase with the use of a device that helps transdermal absorption of the active ingredient [142]. Nicotinamide can be combined with other active ingredients for added skin-lightening efficacy [143]. If nicotinamide plays a role in inhibiting melanosome transfer in preventing skin pigmentation, combining it with other active ingredients that play a different role would maximize the effect. Potential candidates for combination with nicotinamide for this purpose may include substances that inhibit the expression of enzymes involved in melanin synthesis [149,150], that inhibit the catalytic activity of an enzyme [151,152,153], and/or that reduce the relative ratio of eumelanin to pheomelanin [154,155]. 

Although nicotinamide is considered a very safe nutrient, its long-term use at very high doses may cause side effects to the liver or other organs [156]. Serious metabolic and epigenetic changes were observed in rats fed with high doses of nicotinamide over a long period [157]. When 10% nicotinamide was applied to the skin, human subjects did not feel stinging sensation or flushing, and irritation did not appear in a single-use primary irritation test or a cumulative irritation test for 21 days with 5% product [158]. Thus, nicotinamide is well tolerated by the skin at the normally used concentrations (<5%) [159]. Nevertheless, the skin reaction to nicotinamide can vary depending on the skin condition of each individual; thus, it is necessary to consult a doctor if severe side effects exist.

### 9.2. Mechanisms of Skin Antiaging Action of Nicotinamide 

This review highlights the fact that nicotinamide can have a wide range of effects on cellular metabolism; therefore, it is not easy to identify the mechanism of this substance in controlling skin aging. Although nicotinamide, a reaction product of sirtuins or PARPs, can inhibit these enzymes at several tens of micromolar concentrations [160,161], it is unclear whether its intracellular concentrations can reach that high. Nicotinamide is known to be absorbed into cells to some extent, but its transporter has not been identified yet, whereas transporters of nicotinic acid and 1-methylnicotinamide are relatively well known [162]. The different effects of nicotinamide on the lifespans of yeast and mammalian cells [67,68,69] suggest that in the latter case, the intracellular concentration is lower, and thus, the sirtuins inhibitory concentration cannot be reached. A possible cause is that nicotinamide is rapidly converted to NMN by NAMPT outside the cell. In agreement with this assumption, a secreted form of NAMPT exists outside the cell [163], and a transporter responsible for the intracellular uptake of NMN produced by this enzyme was recently discovered [164]. Accordingly, it is emphasized that the biological effects of nicotinamide can be dependent on the activity of NAMPT, which plays an important role in aging [165].

Natural aging and photoaging commonly accompany cellular senescence, chronic inflammation, and changes in the ECM and skin barrier along with external appearance changes. In senescent cells, NAMPT, NAD^+^ pool, and mitochondrial electron transport activity decrease, while ROS production increases [71,73,75]. The supply of nicotinamide helps to normalize these changes, delaying cell senescence and extending its lifespan [72,74,75,77]. Nicotinamide inhibits the production of inflammatory cytokines and PGs in keratinocytes or in three-dimensional skin models that are exposed to UV radiation [58,59]. Nicotinamide decreases ECM-degrading enzymes and increases collagen synthesis in dermal fibroblasts [100,101,102]. Nicotinamide enhances the structural and functional integrity of the skin barrier by increasing the synthesis of lipid components [105]. Although nicotinamide exhibits various biological activities, the evidence for the existence of a specific molecular target is not clear. For now, it is believed that nicotinamide contributes to skin homeostasis by regulating the redox status of cells along with various metabolites produced from it. 

### 9.3. Mechanisms of Skin Depigmenting Action of Nicotinamide

The effects of nicotinamide on melanin synthesis in mono-cultured melanocytes are diverse [109,110]. It is presumed that nicotinamide can help restore intrinsic melanin synthesis in melanocytes when it is impaired for some reason, whereas it can prevent excessive melanin synthesis stimulated by external signals. In the keratinocyte–melanocyte co-culture system, nicotinamide appears to consistently decrease the amount of melanin delivered to keratinocytes [22,111]. This finding has been confirmed in other independent studies [166,167]. Therefore, the interaction between these two cell types can be modulated by nicotinamide, but the underlying molecular mechanism remains to be explored. 

It is worth noting PAR-2 as the main director of melanosome transfer from melanocytes to keratinocytes [122]. The receptor is expressed in keratinocytes, is activated by stimuli, such as UV radiation or hydrogen peroxide, and releases PGE_2_, which warns surrounding cells including melanocytes [124,125]. The melanocytes are then activated by this signal to initiate the synthesis of melanin and biogenesis of melanosomes and deliver mature melanosomes to keratinocytes via dendrites [126,127]. In this scenario, if a substance could affect either or both of these two steps, it would exert an inhibitory effect on skin hyperpigmentation. Studying whether nicotinamide affects the PAR-2 mediated signal transduction process will be important in elucidating the mechanism of the pigmentation inhibitory action of this substance.

A recent study showed that NNT could regulate melanin synthesis in the skin [168]. In human melanoma cells, depletion of NNT increased melanin synthesis, which was inhibited by N-acetyl cysteine. Overexpression of NNT decreased eumelanin synthesis, which was attributed to increases in the NADPH/NADP^+^ ratio and the GSH/GSSG ratio. NNT expression levels were low in post-inflammatory hyperpigmentation or age spots of human skin. It would be interesting to examine the effects of nicotinamide on pigmentation in the skin with altered NNT activity. 

### 9.4. Questions to Be Answered in Future Studies 

As discussed above, important and interesting questions about the mechanism of action and biological activity of nicotinamide remain unclear, with the following questions to list a few:

● Is there any specific molecular target of nicotinamide for the control of skin aging or pigmentation?
● Is the antiaging effect of nicotinamide is due to its intrinsic property or its metabolites?
● How does nicotinamide modulate the cell-to-cell interactions in the skin?
● Whether and how does nicotinamide regulate PAR-2 or NNT involved in skin pigmentation?
● Does nicotinamide supplementation affect the NADPH/NADP^+^ ratio and the GSH/GSSG ratio in the skin?
● Would it be more effective if the dose of nicotinamide is adjusted according to the NAD^+^ pool level, which varies depending on the individual skin condition?
● How does nicotinamide affect the self-renewal, proliferation, differentiation, senescence, and eventual exhaustion of epidermal stem cells?

## 10. Conclusions

The action of nicotinamide in controlling skin aging and pigmentation may be due to the intrinsic properties of nicotinamide, or the properties of other metabolites derived from nicotinamide, or both. Nicotinamide mitigates oxidative stress of cells via a direct ROS/free radical-scavenging action or an indirect action that enhances the antioxidant capacity of cells. Nicotinamide is metabolized by NAMPT to restore the NAD^+^ pool and delay the senescence of cells. Both nicotinamide and its metabolites, such as NMN and 1-methylnicotinamide exert anti-inflammatory properties in various experimental models. Therefore, for cosmetic applications of nicotinamide, it is important to consider the biological activities of its metabolites as well as nicotinamide itself. 

External stimuli, such as UV light and PM, internal stimuli, and chronological time cause skin aging and hyperpigmentation through direct and indirect paths. When epidermal keratinocytes are activated through ROS-mediated signaling, the expressions of inflammatory cytokines, PGs, and other signaling molecules are induced. Signaling molecules, such as stratifin, secreted from keratinocytes activate dermal fibroblasts to release MMPs for ECM remodeling. PGs secreted from keratinocytes activate epidermal melanocytes to increase melanin synthesis and melanosome biogenesis, and promote intercellular melanosome transfer from melanocytes to keratinocytes. These overall processes can be alleviated by supplementation of nicotinamide, resulting in reduced skin aging and pigmentation.

Therefore, nutritional and pharmacological actions of nicotinamide may be mediated by a complex mechanism including several metabolic pathways and multiple signaling processes. Aside from the identity of the mechanism of action, the results of many clinical trials suggest that nicotinamide is a beneficial cosmetic ingredient that helps skin health and beauty without any severe side effects.

## Figures and Tables

**Table 1:** Skin antiaging efficacy of cosmeceuticals containing nicotinamide alone or with other active ingredients.

Literature	Study Format	No. of Subjects	Compared Formulations	Treatment	Key Findings
[23]	A double-blind, placebo-controlled, split-face, left–right randomized clinical study	50	An oil-in-water moisturizer (placebo control	To each side of the face was applied each product, twice daily for 12 weeks.	Improved fine lines/wrinkles, hyperpigmentation spots, texture, red blotchiness, and skin yellowing (sallowness) compared to the control in endpoints.
			5% nicotinamide		
[24]	A double-blind, placebo formulation–controlled, split-face study with left–right randomization	50	An oil-in-water moisturizer (placebo control	To each side of the face was applied each product, twice daily for 12 weeks.	Reduced fine lines, wrinkles, hyperpigmented spots, red blotchiness, and skin sallowness (yellowing), and increased elasticity (as measured via cutometry).
			5% nicotinamide		
[133]	A randomized, double-blind, placebo-controlled, split-face comparative study	27	An aqueous serum	Test serum was applied evenly to one side of the face and vehicle to the other side twice daily for 12 weeks.	Combination of kinetin and nicotinamide reduced pore, wrinkle, unevenness, erythema, and spot at weeks 8 and 12 and increased corneal moisture at week 12. Nicotinamide alone reduced pore and unevenness at week 8 and wrinkle at week 12.
			0.03% kinetin + 4% nicotinamide		
		25	An aqueous serum		
			4% nicotinamide		
[134]	A randomized, placebo-controlled, split-face study	30	A vehicle lotion	The test product was applied on wrinkles of one side and a control product on the other side for 8 weeks.	Test product reduced wrinkle grades and average roughness of skin surface (Ra value) in the tested skin area to lower levels compared to pre-application (*p* < 0.001) and the vehicle control (*p* < 0.001) in endpoints.
			4% nicotinamide		
[135]	A randomized, parallel-group facial appearance study	99	A daytime lotion (SPF 30) containing 5% nicotinamide and peptides; a night cream containing nicotinamide and peptides; a wrinkle treatment containing nicotinamide, peptides, and 0.3% retinyl propionate.	Subjects applied a wrinkle treatment twice daily, a daytime lotion, and the night cream daily.	The cosmetic regimen significantly improved wrinkle appearance after 8 weeks relative to tretinoin in the total population, with comparable benefits in subject cohorts (*n* = 25) who continued treatment for an additional 16 weeks.
		97	0.02% tretinoin in an emollient base; a sunscreen (SPF 30)		
[136]	A randomized, double-blinded, vehicle-controlled, split-face study	40	A simple oil-in-water emulsion	Subjects applied the products twice daily to either the left- or right-hand side of their face at 2 mg cm^−2^.	Test product improved stratum corneum hydration, barrier function, elasticity, and surface topography compared with the vehicle control in endpoints.
			2% gold silk sericin, 5% nicotinamide, 0.1% signaline^TM^ (diacylglycerol and fatty alcohols)		
[137]	An open-label, single-center study	25	0.5% retinol, 4.4% nicotinamide, 1% resveratrol, and 1.1% hexylresorcinol	Treatment at night for 10 weeks.	The formulation improved hyperpigmentation, overall skin clarity, evenness of skin tone, and wrinkles compared to baseline at week 4 and through week 10.
[138]	A double-blind, randomized, split-face, vehicle-controlled study	24	2% human adipocyte-derived mesenchymal stem cell-conditioned medium and 2% nicotinamide	Applied twice daily for 3 weeks after fractional ablative CO_2_ laser treatment.	The formulation reduced the wrinkle index (*p* = 0.036) and melanin index (*p* = 0.043) compared to the control group.
			A vehicle cream		

**Table 2:** Skin-lightening efficacy of cosmeceuticals containing nicotinamide.

Literature	Study Format	No. of Subjects	Compared Formulations	Treatment	Key Findings
[22]	A randomized, split-face, double-blind, paired clinical study	18	5% nicotinamide	Subjects applied a test or a control moisturizer to each side of the face twice daily for 8 weeks.	The side of the face receiving the test moisturizer showed a significant decrease in the total hyperpigmented area measured by image analysis and a reduction in visually assessed hyperpigmentation degree compared to the side receiving the control moisturizer after 4 weeks or 8 weeks of treatment.
			A control moisturizer		
	A randomized, split-face, double-blind, round-robin design	120	A vehicle moisturizer	Applied two of three different products, each product to each side of their face twice daily for 8 weeks.	After 4 weeks, L* value of the treated sides was highest with a test sunscreen moisturizer, followed by a control sunscreen moisturizer, and a vehicle moisturizer.
			A control sunscreen moisturizer (SPF 15)		
			A test sunscreen moisturizer containing 2% nicotinamide		
[111]	A double-blinded, randomized, vehicle-controlled, split-face design human clinical trial	39	5% nicotinamide	Subjects applied either test or control product to the assigned sides of their faces twice a day for 8 weeks.	5% Nicotinamide-containing moisturizer demonstrated a higher reduction in hyperpigmented spot than the vehicle moisturizer, after 4 and 8 weeks of treatment. 2% Nicotinamide did not show a statistically significant effect compared to the vehicle moisturizer.
			A vehicle moisturizer		
		40	2% nicotinamide		
			A vehicle moisturizer		
[139]	A double-blind, randomized, clinical trial	27	4% nicotinamide	Melasma patients applied a product on the left side of the face and the other on the right side for 8 weeks.	After 8 weeks of treatment, MASI score was decreased, L* value was increased and a* value was unchanged by both treatments compared to the baseline values. Good to excellent improvement was observed in 44% of patients receiving nicotinamide and 55% of patients receiving hydroquinone.
			4% hydroquinone		
[140]	A randomized, double-blind, left–right axilla, placebo-controlled trial	24	4% nicotinamide (*n* = 16 axillae)	Treatment at night for 9 weeks.	At 9 weeks, L* values in the nicotinamide and desonide groups were increased more compared with the placebo group. Desonide was more effective than nicotinamide (*p* = 0.002).
			0.05% desonide (*n* = 16 axillae)		
			A placebo cream (*n* = 16 axillae)		

**Table 3:** Skin-lightening efficacy of cosmeceuticals containing nicotinamide in combination with other active ingredients.

Literature	Study Format	No. of Subjects	Compared Formulations	Treatment	Key Findings
[142]	A randomized, split-face design	30	No treatment	Subjects used the ultrasound device for 10 min with or without a gel every night for 4 weeks.	Use of ultrasound treatment with a gel reduced hyperpigmentation compared with no treatment or treatment of a gel alone after 4 weeks.
			Ultrasound treatment		
		30	A gel containing 2% ascorbyl glucoside, and 3.5% nicotinamide		
			Ultrasound treatment with a gel		
[143]	Double-blind, left–right, randomized, split-face clinical studies	40	A vehicle emulsion	Treatment in the morning and evening before bedtime for 8 weeks.	Combination formulation and nicotinamide alone reduced the appearance of hyperpigmentation after 8 weeks. The combination was more effective than nicotinamide alone (*p* = 0.0003).
			5% nicotinamide		
		40	5% nicotinamide		
			5% nicotinamide plus 1% n-undecylenoyl phenylalanine		
[144]	A double-blind, vehicle-controlled, full-face, parallel-group clinical study	101	4% nicotinamide plus 2% N-acetyl glucosamine	Treatment of a sunscreen lotion in the morning and test creams in the evening for 8 weeks.	The formulation reduced the area of facial spots and the appearance of irregular pigmentation at weeks 6 (*p* = 0.0270 and weeks 8 (*p* = 0.037).
		101	A vehicle cream		
[145]	A single-center, randomized, double-blind, controlled study	30	*trans*-4-(amino methyl) cyclohexanecarboxylic acid, potassium azeloyl diglycinate, and nicotinamide	Treatment in the morning and before bedtime for 8 weeks.	The formulation reduced the relative melanin value at week 6 (*p* = 0.006); Reduced MASI scores at week 4 (*p* = 0.005).
		30	Emulsion-based control		
[146]	A prospective, randomized, double-blind, vehicle-controlled clinical study	21	2% nicotinamide plus 2% tranexamic acid	Treatment in the morning and evening for 8 weeks.	The formulation reduced melanin index from baseline at weeks 4 (*p* < 0.001) and 8 (*p* < 0.001). It reduced the mean pigment intensity score compared with the vehicle control formulation (*p* = 0.015).
		21	A vehicle cream		
[147]	A clinical study	55	3% tranexamic acid, 1% kojic acid, 5% nicotinamide, and 5% hydroxyethylpiperazineethane sulfonic acid	Treatment in the morning and evening for 12 weeks.	The formulation reduced melanin index and improved the appearance of hyperpigmentation compared to both pre-treatment baselines.
[148]	A prospective, randomized, controlled split-face study	18	SKNB19 formulation containing epidermal growth factor, tranexamic acid, vitamin C, arbutin, nicotinamide, and other ingredients	Treatment in the morning and night for 8 weeks. Hydroquinone application only nightly.	SKNB19 improved the appearance of hyperpigmentation when compared with 4% hydroquinone.
			Standard formulation containing 4% hydroquinone		

## Abbreviations

ADP	adenosine diphosphate
AMP	adenosine monophosphate
ATP	adenosine triphosphate
cADPR	cyclic ADP ribose
CYP	cytochrome P450
ECM	extracellular matrix
GSH	glutathione
GSSG	glutathione disulfide
IL	interleukin
MAPK	mitogen-activated protein kinase
MASI	melasma area and severity index
MMP	matrix metalloproteinase
NaAD ^+^	nicotinic acid adenine dinucleotide
NaADP ^+^	nicotinic acid adenine dinucleotide phosphate
NAD ^+^	nicotinamide adenine dinucleotide
NADH	reduced nicotinamide adenine dinucleotide
NADP ^+^	nicotinamide adenine dinucleotide phosphate
NADPH	reduced nicotinamide adenine dinucleotide phosphate
NaMN	nicotinic acid mononucleotide
NAMPT	nicotinamide phosphoribosyltransferase
NF-κB	nuclear factor-κB
NMN	nicotinamide mononucleotide
NNT	nicotinamide nucleotide transhydrogenase
PAR-2	protease-activated receptor-2
PARP	poly(ADP-ribose) polymerase
PG	prostaglandin
PM	particulate matter
PPi	inorganic pyrophosphate
PRPP	phosphoribosyl pyrophosphate
ROS	reactive oxygen species
sir2	silent information regulator-2
SIRT1	sirtruin1
SPF	sun protection factor
TGF-β	transforming growth factor-β
TNF-α	tumor necrosis factor-α
UV	ultraviolet

## References

[1] Chambers E.S., Vukmanovic-Stejic M. (2020). Skin barrier immunity and ageing. Immunology.

[2] Fisher G.J., Kang S., Varani J., Bata-Csorgo Z., Wan Y., Datta S., Voorhees J.J. (2002). Mechanisms of photoaging and chronological skin aging. Arch. Dermatol..

[3] Rittie L., Fisher G.J. (2015). Natural and sun-induced aging of human skin. Cold Spring Harb. Perspect. Med..

[4] Tzaphlidou M. (2004). The role of collagen and elastin in aged skin: An image processing approach. Micron.

[5] Pizzino G., Irrera N., Cucinotta M., Pallio G., Mannino F., Arcoraci V., Squadrito F., Altavilla D., Bitto A. (2017). Oxidative Stress: Harms and Benefits for Human Health. Oxid. Med. Cell. Longev..

[6] Gu Y., Han J., Jiang C., Zhang Y. (2020). Biomarkers, oxidative stress and autophagy in skin aging. Ageing Res. Rev..

[7] Kammeyer A., Luiten R.M. (2015). Oxidation events and skin aging. Ageing Res. Rev..

[8] Shah A.A., Sinha A.A. (2013). Oxidative stress and autoimmune skin disease. Eur. J. Dermatol..

[9] Bickers D.R., Athar M. (2006). Oxidative stress in the pathogenesis of skin disease. J. Investig. Dermatol..

[10] Awad F., Assrawi E., Louvrier C., Jumeau C., Giurgea I., Amselem S., Karabina S.A. (2018). Photoaging and skin cancer: Is the inflammasome the missing link?. Mech. Ageing Dev..

[11] Boo Y.C. (2020). Natural Nrf2 Modulators for Skin Protection. Antioxidants.

[12] Baek J., Lee M.G. (2016). Oxidative stress and antioxidant strategies in dermatology. Redox Rep..

[13] Hegyi J., Schwartz R.A., Hegyi V. (2004). Pellagra: Dermatitis, dementia, and diarrhea. Int. J. Dermatol..

[14] Kirkland J.B. (2009). Niacin Status, NAD Distribution and ADP-Ribose Metabolism. Curr. Pharm. Des..

[15] De Figueiredo L.F., Gossmann T.I., Ziegler M., Schuster S. (2011). Pathway analysis of NAD^+^ metabolism. Biochem. J..

[16] Mattiussi A.J., Blais D. (1992). Niacin Versus Niacinamide. Can. Med Assoc. J..

[17] MacKay D., Hathcock J., Guarneri E. (2012). Niacin: Chemical forms, bioavailability, and health effects. Nutr. Rev..

[18] Surjana D., Damian D.L. (2011). Nicotinamide in dermatology and photoprotection. Skinmed.

[19] Forbat E., Al-Niaimi F., Ali F.R. (2017). Use of nicotinamide in dermatology. Clin. Exp. Dermatol..

[20] Ballotti R., Healy E., Bertolotto C. (2019). Nicotinamide as a chemopreventive therapy of skin cancers. Too much of good thing?. Pigment. Cell Melanoma Res..

[21] Snaidr V.A., Damian D.L., Halliday G.M. (2019). Nicotinamide for photoprotection and skin cancer chemoprevention: A review of efficacy and safety. Exp. Dermatol..

[22] Hakozaki T., Minwalla L., Zhuang J., Chhoa M., Matsubara A., Miyamoto K., Greatens A., Hillebrand G.G., Bissett D.L., Boissy R.E. (2002). The effect of niacinamide on reducing cutaneous pigmentation and suppression of melanosome transfer. Br. J. Dermatol..

[23] Bissett D.L., Miyamoto K., Sun P., Li J., Berge C.A. (2004). Topical niacinamide reduces yellowing, wrinkling, red blotchiness, and hyperpigmented spots in aging facial skin. Int. J. Cosmet. Sci..

[24] Bissett D.L., Oblong J.E., Berge C.A. (2005). Niacinamide: A B vitamin that improves aging facial skin appearance. Dermatol. Surg..

[25] Otte N., Borelli C., Korting H.C. (2005). Nicotinamide—Biologic actions of an emerging cosmetic ingredient. Int. J. Cosmet. Sci..

[26] Braidy N., Berg J., Clement J., Khorshidi F., Poljak A., Jayasena T., Grant R., Sachdev P. (2019). Role of Nicotinamide Adenine Dinucleotide and Related Precursors as Therapeutic Targets for Age-Related Degenerative Diseases: Rationale, Biochemistry, Pharmacokinetics, and Outcomes. Antioxid. Redox Signal..

[27] Fukuwatari T., Shibata K. (2013). Nutritional aspect of tryptophan metabolism. Int. J. Tryptophan Res..

[28] Shibata K. (2018). Organ Co-Relationship in Tryptophan Metabolism and Factors That Govern the Biosynthesis of Nicotinamide from Tryptophan. J. Nutr. Sci. Vitaminol..

[29] Kennedy B.E., Sharif T., Martell E., Dai C., Kim Y., Lee P.W.K., Gujar S.A. (2016). NAD^+^ salvage pathway in cancer metabolism and therapy. Pharmacol. Res..

[30] Tedeschi P.M., Bansal N., Kerrigan J.E., Abali E.E., Scotto K.W., Bertino J.R. (2016). NAD^+^ Kinase as a Therapeutic Target in Cancer. Clin. Cancer Res..

[31] Zhang Q., Padayatti P.S., Leung J.H. (2017). Proton-Translocating Nicotinamide Nucleotide Transhydrogenase: A Structural Perspective. Front. Physiol..

[32] Chini C.C.S., Zeidler J.D., Kashyap S., Warner G., Chini E.N. (2021). Evolving concepts in NAD^+^ metabolism. Cell Metab..

[33] Schweiker S.S., Tauber A.L., Sherry M.E., Levonis S.M. (2018). Structure, Function and Inhibition of Poly(ADP-ribose)polymerase, Member 14 (PARP14). Mini Rev. Med. Chem..

[34] Palazzo L., Mikolcevic P., Mikoc A., Ahel I. (2019). ADP-ribosylation signalling and human disease. Open Biol..

[35] Hassa P.O., Haenni S.S., Elser M., Hottiger M.O. (2006). Nuclear ADP-ribosylation reactions in mammalian cells: Where are we today and where are we going?. Microbiology and Molecular Biology Reviews.

[36] Morales J.C., Li L.S., Fattah F.J., Dong Y., Bey E.A., Patel M., Geo J.M., Boothman D.A. (2014). Review of Poly (ADP-ribose) Polymerase (PARP) Mechanisms of Action and Rationale for Targeting in Cancer and Other Diseases. Crit. Rev. Eukaryot. Gene Expr..

[37] Watroba M., Dudek I., Skoda M., Stangret A., Rzodkiewicz P., Szukiewicz D. (2017). Sirtuins, epigenetics and longevity. Ageing Res. Rev..

[38] Klein M.A., Denu J.M. (2020). Biological and catalytic functions of sirtuin 6 as targets for small-molecule modulators. J. Biol. Chem..

[39] Chang H.C., Guarente L. (2014). SIRT1 and other sirtuins in metabolism. Trends Endocrinol. Metab..

[40] Kar A., Mehrotra S., Chatterjee S. (2020). CD38: T Cell Immuno-Metabolic Modulator. Cells.

[41] Aarhus R., Graeff R.M., Dickey D.M., Walseth T.F., Lee H.C. (1995). ADP-ribosyl cyclase and CD38 catalyze the synthesis of a calcium-mobilizing metabolite from NADP^+^. J. Biol. Chem..

[42] Nam T.S., Park D.R., Rah S.Y., Woo T.G., Chung H.T., Brenner C., Kim U.H. (2020). Interleukin-8 drives CD38 to form NAADP from NADP^+^ and NAAD in the endolysosomes to mobilize Ca^2+^ and effect cell migration. FASEB J..

[43] Yu P.L., Cai X.B., Liang Y., Wang M.X., Yang W. (2020). Roles of NAD^+^ and Its Metabolites Regulated Calcium Channels in Cancer. Molecules.

[44] Gul R., Park D.R., Shawl A.I., Im S.Y., Nam T.S., Lee S.H., Ko J.K., Jang K.Y., Kim D., Kim U.H. (2016). Nicotinic Acid Adenine Dinucleotide Phosphate (NAADP) and Cyclic ADP-Ribose (cADPR) Mediate Ca^2+^ Signaling in Cardiac Hypertrophy Induced by beta-Adrenergic Stimulation. PLoS ONE.

[45] Pissios P. (2017). Nicotinamide N-Methyltransferase: More Than a Vitamin B3 Clearance Enzyme. Trends Endocrinol. Metab..

[46] Felsted R.L., Chaykin S. (1967). N1-methylnicotinamide oxidation in a number of mammals. J. Biol. Chem..

[47] Real A.M., Hong S.Y., Pissios P. (2013). Nicotinamide N-Oxidation by CYP2E1 in Human Liver Microsomes. Drug Metab. Dispos..

[48] Nadzhimutdinov K.N., Mavlianov I.R., Umarov E.F., Mutalov N.K. (1993). The effect of alpha-tocopherol and nicotinamide on lipid peroxidation and the activity of the antioxidant system in the lung tissue of premature rat pups. Eksp. Klin. Farmakol..

[49] Legon’kova L.F., Bushma M.I., Zverinskii I.V., Abakumov G.Z., Zavodnik L.V. (1997). The effect of nicotinamide, methionine and alpha-tocopherol on the liver conjugating and mono-oxygenase systems and on lipid peroxidation in hepatosis-hepatitis in rats. Eksp. Klin. Farmakol..

[50] Velykyi M.M., Burda V.A., Biront N.V., Oliiarnyk O.D., Velykyi A.M. (1996). The effect of nicotinamide on the enzymatic activity of the antioxidant defense in experimental diabetes. Ukr. Biokhimicheskii Zhurnal (1978).

[51] Kamat J.P., Devasagayam T.P. (1996). Methylene blue plus light-induced lipid peroxidation in rat liver microsomes: Inhibition by nicotinamide (vitamin B3) and other antioxidants. Chem. Biol. Interact..

[52] Kamat J.P., Devasagayam T.P.A. (1999). Nicotinamide (vitamin B-3) as an effective antioxidant against oxidative damage in rat brain mitochondria. Redox Rep..

[53] Izdebska M., Halas-Wisniewska M., Adamczyk I., Lewandowska I., Kwiatkowska I., Gagat M., Grzanka A. (2018). The protective effect of niacinamide on CHO AA8 cell line against ultraviolet radiation in the context of main cytoskeletal proteins. Adv. Clin. Exp. Med..

[54] Chhabra G., Garvey D.R., Singh C.K., Mintie C.A., Ahmad N. (2019). Effects and Mechanism of Nicotinamide Against UVA- and/or UVB-mediated DNA Damages in Normal Melanocytes. Photochem. Photobiol..

[55] Zhen A.X., Piao M.J., Kang K.A., Fernando P.D.S.M., Kang H.K., Koh Y.S., Yi J.M., Hyun J.W. (2019). Niacinamide Protects Skin Cells from Oxidative Stress Induced by Particulate Matter. Biomol. Ther..

[56] Mi T.Y., Dong Y.Y., Santhanam U., Huang N. (2019). Niacinamide and 12-hydroxystearic acid prevented benzo(a)pyrene and squalene peroxides induced hyperpigmentation in skin equivalent. Exp. Dermatol..

[57] Grange P.A., Raingeaud J., Calvez V., Dupin N. (2009). Nicotinamide inhibits Propionibacterium acnes-induced IL-8 production in keratinocytes through the NF-kappaB and MAPK pathways. J. Dermatol. Sci..

[58] Monfrecola G., Gaudiello F., Cirillo T., Fabbrocini G., Balato A., Lembo S. (2013). Nicotinamide downregulates gene expression of interleukin-6, interleukin-10, monocyte chemoattractant protein-1, and tumour necrosis factor-alpha gene expression in HaCaT keratinocytes after ultraviolet B irradiation. Clin. Exp. Dermatol..

[59] Bierman J.C., Laughlin T., Tamura M., Hulette B., Mack C.E., Sherrill J.D., Tan C.Y.R., Morenc M., Bellanger S., Oblong J.E. (2020). Niacinamide mitigates SASP-related inflammation induced by environmental stressors in human epidermal keratinocytes and skin. Int. J. Cosmet. Sci..

[60] Bryniarski K., Biedron R., Jakubowski A., Chlopicki S., Marcinkiewicz J. (2008). Anti-inflammatory effect of 1-methylnicotinamide in contact hypersensitivity to oxazolone in mice; involvement of prostacyclin. Eur. J. Pharmacol..

[61] Biedron R., Ciszek M., Tokarczyk M., Bobek M., Kurnyta M., Slominska E.M., Smolenski R.T., Marcinkiewicz J. (2008). 1-Methylnicotinamide and nicotinamide: Two related anti-inflammatory agents that differentially affect the functions of activated macrophages. Arch. Immunol. Ther. Exp..

[62] Wozniacka A., Wieczorkowska M., Gebicki J., Sysa-Jedrzejowska A. (2005). Topical application of 1-methylnicotinamide in the treatment of rosacea: A pilot study. Clin. Exp. Dermatol..

[63] Pietrzak L., Mogielnicki A., Buczko W. (2009). Nicotinamide and its metabolite N-methylnicotinamide increase skin vascular permeability in rats. Clin. Exp. Dermatol..

[64] Jiang N., Wang M., Song J.Y., Liu Y.G., Chen H., Mu D., Xia M. (2016). N-methylnicotinamide protects against endothelial dysfunction and attenuates atherogenesis in apolipoprotein E-deficient mice. Mol. Nutr. Food Res..

[65] Huynh P.K., Wilder J., Hiller S., Hagaman J., Takahashi N., Maeda-Smithies N., Li F. (2020). Beneficial effects of nicotinamide on hypertensive mice with impaired endothelial nitric oxide function. J. Exp. Nephrol..

[66] Zhou X.R., Du H.H., Ni L.Y., Ran J., Hu J., Yu J.J., Zhao X. (2021). Nicotinamide Mononucleotide Combined with Lactobacillus fermentum TKSN041 Reduces the Photoaging Damage in Murine Skin by Activating AMPK Signaling Pathway. Front. Pharmacol..

[67] Anderson R.M., Bitterman K.J., Wood J.G., Medvedik O., Sinclair D.A. (2003). Nicotinamide and PNC1 govern lifespan extension by calorie restriction in Saccharomyces cerevisiae. Nature.

[68] Matuoka K., Chen K.Y., Takenawa T. (2001). Rapid reversion of aging phenotypes by nicotinamide through possible modulation of histone acetylation. Cell. Mol. Life Sci..

[69] Lim C.S., Potts M., Helm R.E. (2006). Nicotinamide extends the replicative life span of primary human cells. Mech. Ageing Dev..

[70] Porcu M., Chiarugi A. (2005). The emerging therapeutic potential of sirtuin-interacting drugs: From cell death to lifespan extension. Trends Pharmacol. Sci..

[71] Adams J.D., Klaidman L.K. (2007). Sirtuins, nicotinamide and aging: A critical review. Lett. Drug Des. Discov..

[72] Massudi H., Grant R., Braidy N., Guest J., Farnsworth B., Guillemin G.J. (2012). Age-Associated Changes In Oxidative Stress and NAD^+^ Metabolism In Human Tissue. PLoS ONE.

[73] Oblong J.E. (2014). The evolving role of the NAD plus/nicotinamide metabolome in skin homeostasis, cellular bioenergetics, and aging. DNA Repair.

[74] Kang H.T., Il Lee H., Hwang E.S. (2006). Nicotinamide extends replicative lifespan of human cells. Aging Cell.

[75] Kwak J.Y., Ham H.J., Kim C.M., Hwang E.S. (2015). Nicotinamide exerts antioxidative effects on senescent cells. Mol. Cells.

[76] Oblong J.E., Bowman A., Rovito H.A., Jarrold B.B., Sherrill J.D., Black M.R., Nelson G., Kimball A.B., Birch-Machin M.A. (2020). Metabolic dysfunction in human skin: Restoration of mitochondrial integrity and metabolic output by nicotinamide (niacinamide) in primary dermal fibroblasts from older aged donors. Aging Cell.

[77] Borradaile N.M., Pickering J.G. (2009). Nicotinamide phosphoribosyltransferase imparts human endothelial cells with extended replicative lifespan and enhanced angiogenic capacity in a high glucose environment. Aging Cell.

[78] Rovito H.A., Oblong J.E. (2013). Nicotinamide preferentially protects glycolysis in dermal fibroblasts under oxidative stress conditions. Br. J. Dermatol..

[79] Khaidizar F.D., Nakahata Y., Kume A., Sumizawa K., Kohno K., Matsui T., Bessho Y. (2017). Nicotinamide phosphoribosyltransferase delays cellular senescence by upregulating SIRT1 activity and antioxidant gene expression in mouse cells. Genes Cells.

[80] Tan C.L., Chin T., Tan C.Y.R., Rovito H.A., Quek L.S., Oblong J.E., Bellanger S. (2019). Nicotinamide Metabolism Modulates the Proliferation/Differentiation Balance and Senescence of Human Primary Keratinocytes. J. Investig. Dermatol..

[81] Taub A.F., Pham K. (2018). Stem Cells in Dermatology and Anti-aging Care of the Skin. Facial Plast. Surg. Clin. N. Am..

[82] Zouboulis C.C., Adjaye J., Akamatsu H., Moe-Behrens G., Niemann C. (2008). Human skin stem cells and the ageing process. Exp. Gerontol..

[83] Liu N., Matsumura H., Kato T., Ichinose S., Takada A., Namiki T., Asakawa K., Morinaga H., Mohri Y., De Arcangelis A. (2019). Stem cell competition orchestrates skin homeostasis and ageing. Nature.

[84] Gannon H.S., Donehower L.A., Lyle S., Jones S.N. (2011). Mdm2-p53 signaling regulates epidermal stem cell senescence and premature aging phenotypes in mouse skin. Dev. Biol..

[85] Chu G.Y., Chen Y.F., Chen H.Y., Chan M.H., Gau C.S., Weng S.M. (2018). Stem cell therapy on skin: Mechanisms, recent advances and drug reviewing issues. J. Food Drug Anal..

[86] Grabowska W., Sikora E., Bielak-Zmijewska A. (2017). Sirtuins, a promising target in slowing down the ageing process. Biogerontology.

[87] Mayack B.K., Sippl W., Ntie-Kang F. (2020). Natural Products as Modulators of Sirtuins. Molecules.

[88] Meng Y., Ren Z., Xu F., Zhou X., Song C., Wang V.Y., Liu W., Lu L., Thomson J.A., Chen G. (2018). Nicotinamide Promotes Cell Survival and Differentiation as Kinase Inhibitor in Human Pluripotent Stem Cells. Stem Cell Rep..

[89] Cole M.A., Quan T., Voorhees J.J., Fisher G.J. (2018). Extracellular matrix regulation of fibroblast function: Redefining our perspective on skin aging. J. Cell Commun. Signal..

[90] Shin J.W., Kwon S.H., Choi J.Y., Na J.I., Huh C.H., Choi H.R., Park K.C. (2019). Molecular Mechanisms of Dermal Aging and Antiaging Approaches. Int. J. Mol. Sci..

[91] Quan T.H., Fisher G.J. (2015). Role of Age-Associated Alterations of the Dermal Extracellular Matrix Microenvironment in Human Skin Aging: A Mini-Review. Gerontology.

[92] Imokawa G., Ishida K. (2015). Biological mechanisms underlying the ultraviolet radiation-induced formation of skin wrinkling and sagging I: Reduced skin elasticity, highly associated with enhanced dermal elastase activity, triggers wrinkling and sagging. Int. J. Mol. Sci..

[93] Ghahary A., Marcoux Y., Karimi-Busheri F., Li Y., Tredget E.E., Kilani R.T., Lam E., Weinfeld M. (2005). Differentiated keratinocyte-releasable stratifin (14-3-3 sigma) stimulates MMP-1 expression in dermal fibroblasts. J. Investig. Dermatol..

[94] Lam E., Kilani R.T., Li Y., Tredget E.E., Ghahary A. (2005). Stratifin-induced matrix metalloproteinase-1 in fibroblast is mediated by c-fos and p38 mitogen-activated protein kinase activation. J. Investig. Dermatol..

[95] Adachi H., Murakami Y., Tanaka H., Nakata S. (2014). Increase of stratifin triggered by ultraviolet irradiation is possibly related to premature aging of human skin. Exp. Dermatol..

[96] Seok J.K., Boo Y.C. (2015). p-Coumaric Acid Attenuates UVB-Induced Release of Stratifin from Keratinocytes and Indirectly Regulates Matrix Metalloproteinase 1 Release from Fibroblasts. Korean J. Physiol. Pharmacol..

[97] Taylor K.R., Gallo R.L. (2006). Glycosaminoglycans and their proteoglycans: Host-associated molecular patterns for initiation and modulation of inflammation. FASEB J..

[98] Frantz C., Stewart K.M., Weaver V.M. (2010). The extracellular matrix at a glance. J. Cell Sci..

[99] Lee D.H., Oh J.H., Chung J.H. (2016). Glycosaminoglycan and proteoglycan in skin aging. J. Dermatol. Sci..

[100] Ratcliffe D.R., Iqbal J., Hussain M.M., Cramer E.B. (2009). Fibrillar collagen type I stimulation of apolipoprotein B secretion in Caco-2 cells is mediated by beta1 integrin. Biochim. Biophys. Acta.

[101] Philips N., Chalensouk-Khaosaat J., Gonzalez S. (2018). Simulation of the Elastin and Fibrillin in Non-Irradiated or UVA Radiated Fibroblasts, and Direct Inhibition of Elastase or Matrix Metalloptoteinases Activity by Nicotinamide or Its Derivatives. J. Cosmet. Sci..

[102] Wessels Q., Pretorius E., Smith C.M., Nel H. (2014). The potential of a niacinamide dominated cosmeceutical formulation on fibroblast activity and wound healing in vitro. Int. Wound J..

[103] Ashkani Esfahani S., Khoshneviszadeh M., Namazi M.R., Noorafshan A., Geramizadeh B., Nadimi E., Razavipour S.T. (2015). Topical Nicotinamide Improves Tissue Regeneration in Excisional Full-Thickness Skin Wounds: A Stereological and Pathological Study. Trauma Mon..

[104] Choi E.H. (2019). Aging of the skin barrier. Clin. Dermatol..

[105] Tanno O., Ota Y., Kitamura N., Katsube T., Inoue S. (2000). Nicotinamide increases biosynthesis of ceramides as well as other stratum corneum lipids to improve the epidermal permeability barrier. Br. J. Dermatol..

[106] Bissett D. (2002). Topical niacinamide and barrier enhancement. Cutis.

[107] Draelos Z.D., Ertel K., Berge C. (2005). Niacinamide-containing facial moisturizer improves skin barrier and benefits subjects with rosacea. Cutis.

[108] Jacobson E.L., Kim H., Kim M., Williams J.D., Coyle D.L., Coyle W.R., Grove G., Rizer R.L., Stratton M.S., Jacobson M.K. (2007). A topical lipophilic niacin derivative increases NAD, epidermal differentiation and barrier function in photodamaged skin. Exp. Dermatol..

[109] Virador V.M., Kobayashi N., Matsunaga J., Hearing V.J. (1999). A standardized protocol for assessing regulators of pigmentation. Anal. Biochem..

[110] Lei T.C., Virador V.M., Vieira W.D., Hearing V.J. (2002). A melanocyte-keratinocyte coculture model to assess regulators of pigmentation in vitro. Anal. Biochem..

[111] Greatens A., Hakozaki T., Koshoffer A., Epstein H., Schwemberger S., Babcock G., Bissett D., Takiwaki H., Arase S., Wickett R.R. (2005). Effective inhibition of melanosome transfer to keratinocytes by lectins and niacinamide is reversible. Exp. Dermatol..

[112] Seiberg M. (2001). Keratinocyte-melanocyte interactions during melanosome transfer. Pigment Cell Res..

[113] Boissy R.E. (2003). Melanosome transfer to and translocation in the keratinocyte. Exp. Dermatol..

[114] Hearing V.J. (2007). Regulating melanosome transfer: Who’s driving the bus?. Pigment Cell Res..

[115] Wu X.F., Hammer J.A. (2014). Melanosome transfer: It is best to give and receive. Curr. Opin. Cell Biol..

[116] Scott G., Leopardi S., Printup S., Madden B.C. (2002). Filopodia are conduits for melanosome transfer to keratinocytes. J. Cell Sci..

[117] Ando H., Niki Y., Ito M., Akiyama K., Matsui M.S., Yarosh D.B., Ichihashi M. (2012). Melanosomes Are Transferred from Melanocytes to Keratinocytes through the Processes of Packaging, Release, Uptake, and Dispersion. J. Investig. Dermatol..

[118] Wu X.F.S., Masedunskas A., Weigert R., Copeland N.G., Jenkins N.A., Hammer J.A. (2012). Melanoregulin regulates a shedding mechanism that drives melanosome transfer from melanocytes to keratinocytes. Proc. Natl. Acad. Sci. USA.

[119] Tarafder A.K., Bolasco G., Correia M.S., Pereira F.J.C., Iannone L., Hume A.N., Kirkpatrick N., Picardo M., Torrisi M.R., Rodrigues I.P. (2014). Rab11b Mediates Melanin Transfer between Donor Melanocytes and Acceptor Keratinocytes via Coupled Exo/Endocytosis. J. Investig. Dermatol..

[120] Santulli R.J., Derian C.K., Darrow A.L., Tomko K.A., Eckardt A.J., Seiberg M., Scarborough R.M., Andradegordon P. (1995). Evidence for the Presence of a Protease-Activated Receptor Distinct from the Thrombin Receptor in Human Keratinocytes. Proc. Natl. Acad. Sci. USA.

[121] Derian C.K., Eckardt A.J., Andrade-Gordon P. (1997). Differential regulation of human keratinocyte growth and differentiation by a novel family of protease-activated receptors. Cell Growth Differ..

[122] Seiberg M., Paine C., Sharlow E., Andrade-Gordon P., Costanzo M., Eisinger M., Shapiro S.S. (2000). The protease-activated receptor 2 regulates pigmentation via keratinocyte-melanocyte interactions. Exp. Cell Res..

[123] Seiberg M., Paine C., Sharlow E., Andrade-Gordon P., Costanzo M., Eisinger M., Shapiro S.S. (2000). Inhibition of melanosome transfer results in skin lightening. J. Investig. Dermatol..

[124] Scott G., Deng A., Rodriguez-Burford C., Seiberg M., Han R.J., Babiarz L., Grizzle W., Bell W., Pentland A. (2001). Protease-activated receptor 2, a receptor involved in melanosome transfer, is upregulated in human skin by ultraviolet irradiation. J. Investig. Dermatol..

[125] Tang L.Y., Li J., Lin X., Wu W.Y., Kang K.F., Fu W.W. (2012). Oxidation Levels Differentially Impact Melanocytes: Low versus High Concentration of Hydrogen Peroxide Promotes Melanin Synthesis and Melanosome Transfer. Dermatology.

[126] Scott G., Leopardi S., Printup S., Malhi N., Seiberg M., Lapoint R. (2004). Proteinase-activated receptor-2 stimulates prostaglandin production in keratinocytes: Analysis of prostaglandin receptors on human melanocytes and effects of PGE2 and PGF2alpha on melanocyte dendricity. J. Investig. Dermatol..

[127] Ma H.J., Ma H.Y., Yang Y., Li P.C., Zi S.X., Jia C.Y., Chen R. (2014). alpha-Melanocyte stimulating hormone (MSH) and prostaglandin E2 (PGE2) drive melanosome transfer by promoting filopodia delivery and shedding spheroid granules: Evidences from atomic force microscopy observation. J. Dermatol. Sci..

[128] Choi E.J., Kang Y.G., Kim J., Hwang J.K. (2011). Macelignan Inhibits Melanosome Transfer Mediated by Protease-Activated Receptor-2 in Keratinocytes. Biol. Pharm. Bull..

[129] Ni J., Wang N., Gao L.L., Li L.L., Zheng S.W., Liu Y.J., Ozukum M., Nikiforova A., Zhao G.M., Song Z.Q. (2016). The effect of the NMDA receptor-dependent signaling pathway on cell morphology and melanosome transfer in melanocytes. J. Dermatol. Sci..

[130] Hu Q.M., Yi W.J., Su M.Y., Jiang S., Xu S.Z., Lei T.C. (2017). Induction of retinal-dependent calcium influx in human melanocytes by UVA or UVB radiation contributes to the stimulation of melanosome transfer. Cell Prolif..

[131] Koike S., Yamasaki K., Yamauchi T., Inoue M., Shimada-Ohmori R., Tsuchiyama K., Aiba S. (2018). Toll-like receptors 2 and 3 enhance melanogenesis and melanosome transport in human melanocytes. Pigment Cell Melanoma Res..

[132] Pillaiyar T., Manickam M., Jung S.H. (2017). Recent development of signaling pathways inhibitors of melanogenesis. Cell. Signal..

[133] Chiu P.C., Chan C.C., Lin H.M., Chiu H.C. (2007). The clinical anti-aging effects of topical kinetin and niacinamide in Asians: A randomized, double-blind, placebo-controlled, split-face comparative trial. J. Cosmet. Dermatol..

[134] Kawada A., Konishi N., Oiso N., Kawara S., Date A. (2008). Evaluation of anti-wrinkle effects of a novel cosmetic containing niacinamide. J. Dermatol..

[135] Fu J.J.J., Hillebrand G.G., Raleigh P., Li J., Marmor M.J., Bertucci V., Grimes P.E., Mandy S.H., Perez M.I., Weinkle S.H. (2010). A randomized, controlled comparative study of the wrinkle reduction benefits of a cosmetic niacinamide/peptide/retinyl propionate product regimen vs. a prescription 0.02% tretinoin product regimen. Br. J. Dermatol..

[136] Berardesca E., Ardigo M., Cameli N., Mariano M., Agozzino M., Matts P.J. (2015). Randomized, double-blinded, vehicle-controlled, split-face study to evaluate the effects of topical application of a Gold Silk Sericin/Niacinamide/Signaline complex on biophysical parameters related to skin ageing. Int. J. Cosmet. Sci..

[137] Farris P., Zeichner J., Berson D. (2016). Efficacy and Tolerability of a Skin Brightening/Anti-Aging Cosmeceutical Containing Retinol 0.5%, Niacinamide, Hexylresorcinol, and Resveratrol. J. Drugs Dermatol..

[138] Lee Y.I., Kim S., Kim J., Kim J., Chung K.B., Lee J.H. (2021). Randomized controlled study for the anti-aging effect of human adipocyte-derived mesenchymal stem cell media combined with niacinamide after laser therapy. J. Cosmet. Dermatol..

[139] Navarrete-Solis J., Castanedo-Cazares J.P., Torres-Alvarez B., Oros-Ovalle C., Fuentes-Ahumada C., Gonzalez F.J., Martinez-Ramirez J.D., Moncada B. (2011). A Double-Blind, Randomized Clinical Trial of Niacinamide 4% versus Hydroquinone 4% in the Treatment of Melasma. Dermatol. Res. Pract..

[140] Castanedo-Cazares J.P., Larraga-Pinones G., Ehnis-Perez A., Fuentes-Ahumada C., Oros-Ovalle C., Smoller B.R., Torres-Alvarez B. (2013). Topical niacinamide 4% and desonide 0.05% for treatment of axillary hyperpigmentation: A randomized, double-blind, placebo-controlled study. Clin. Cosmet. Investig. Dermatol..

[141] Pierard G.E. (1998). EEMCO guidance for the assessment of skin colour. J Eur Acad Dermatol Venereol.

[142] Hakozaki T., Takiwaki H., Miyamoto K., Sato Y., Arase S. (2006). Ultrasound enhanced skin-lightening effect of vitamin C and niacinamide. Skin Res. Technol..

[143] Bissett D.L., Robinson L.R., Raleigh P.S., Miyamoto K., Hakozaki T., Li J., Kelm G.R. (2009). Reduction in the appearance of facial hyperpigmentation by topical N-undecyl-10-enoyl-L-phenylalanine and its combination with niacinamide. J. Cosmet. Dermatol..

[144] Kimball A.B., Kaczvinsky J.R., Li J., Robinson L.R., Matts P.J., Berge C.A., Miyamoto K., Bissett D.L. (2010). Reduction in the appearance of facial hyperpigmentation after use of moisturizers with a combination of topical niacinamide and N-acetyl glucosamine: Results of a randomized, double-blind, vehicle-controlled trial. Br. J. Dermatol..

[145] Viyoch J., Tengamnuay I., Phetdee K., Tuntijarukorn P., Waranuch N. (2010). Effects of Trans-4-(Aminomethyl) Cyclohexanecarboxylic Acid/Potassium Azeloyl Diglycinate/Niacinamide Topical Emulsion in Thai Adults With Melasma: A Single-Center, Randomized, Double-Blind, Controlled Study. Curr. Ther. Res. Clin. Exp..

[146] Lee D.H., Oh I.Y., Koo K.T., Suk J.M., Jung S.W., Park J.O., Kim B.J., Choi Y.M. (2014). Reduction in facial hyperpigmentation after treatment with a combination of topical niacinamide and tranexamic acid: A randomized, double-blind, vehicle-controlled trial. Skin Res. Technol..

[147] Desai S., Ayres E., Bak H., Manco M., Lynch S., Raab S., Du A., Green D., Skobowiat C., Wangari-Talbot J. (2019). Effect of a Tranexamic Acid, Kojic Acid, and Niacinamide Containing Serum on Facial Dyschromia: A Clinical Evaluation. J. Drugs Dermatol..

[148] Kalasho B.D., Minokadeh A., Zhang-Nunes S., Zoumalan R.A., Shemirani N.L., Waldman A.R., Pletzer V., Zoumalan C.I. (2020). Evaluating the Safety and Efficacy of a Topical Formulation Containing Epidermal Growth Factor, Tranexamic Acid, Vitamin C, Arbutin, Niacinamide and Other Ingredients as Hydroquinone 4% Alternatives to Improve Hyperpigmentation: A Prospective, Randomized, Controlled Split Face Study. J. Cosmet. Sci..

[149] Kim J.H., Seok J.K., Kim Y.M., Boo Y.C. (2019). Identification of small peptides and glycinamide that inhibit melanin synthesis using a positional scanning synthetic peptide combinatorial library. Br. J. Dermatol..

[150] Boo Y.C. (2020). Up- or Downregulation of Melanin Synthesis Using Amino Acids, Peptides, and Their Analogs. Biomedicines.

[151] Boo Y.C. (2019). p-Coumaric Acid as An Active Ingredient in Cosmetics: A Review Focusing on its Antimelanogenic Effects. Antioxidants.

[152] Boo Y.C. (2019). Human Skin Lightening Efficacy of Resveratrol and Its Analogs: From in Vitro Studies to Cosmetic Applications. Antioxidants.

[153] Boo Y.C. (2021). Arbutin as a Skin Depigmenting Agent with Antimelanogenic and Antioxidant Properties. Antioxidants.

[154] Chung B.Y., Choi S.R., Moon I.J., Park C.W., Kim Y.H., Chang S.E. (2016). The Glutathione Derivative, GSH Monoethyl Ester, May Effectively Whiten Skin but GSH Does Not. Int. J. Mol. Sci..

[155] Lee H.K., Ha J.W., Hwang Y.J., Boo Y.C. (2021). Identification of L-Cysteinamide as a Potent Inhibitor of Tyrosinase-Mediated Dopachrome Formation and Eumelanin Synthesis. Antioxidants.

[156] Hwang E.S., Song S.B. (2020). Possible Adverse Effects of High-Dose Nicotinamide: Mechanisms and Safety Assessment. Biomolecules.

[157] Li D., Tian Y.J., Guo J., Sun W.P., Lun Y.Z., Guo M., Luo N., Cao Y., Cao J.M., Gong X.J. (2013). Nicotinamide supplementation induces detrimental metabolic and epigenetic changes in developing rats. Br. J. Nutr..

[158] Cosmetic Ingredient Review Expert Panel (2005). Final report of the safety assessment of niacinamide and niacin. Int. J. Toxicol..

[159] Wohlrab J., Kreft D. (2014). Niacinamide—Mechanisms of Action and Its Topical Use in Dermatology. Skin Pharmacol. Physiol..

[160] Guan X.Y., Lin P., Knoll E., Chakrabarti R. (2014). Mechanism of Inhibition of the Human Sirtuin Enzyme SIRT3 by Nicotinamide: Computational and Experimental Studies. PLoS ONE.

[161] Banasik M., Stedeford T., Strosznajder R.P. (2012). Natural Inhibitors of Poly(ADP-ribose) Polymerase-1. Mol. Neurobiol..

[162] Mathialagan S., Bi Y.A., Costales C., Kalgutkar A.S., Rodrigues A.D., Varma M.V.S. (2020). Nicotinic acid transport into human liver involves organic anion transporter 2 (SLC22A7). Biochem. Pharmacol..

[163] Grolla A.A., Torretta S., Gnemmi I., Amoruso A., Orsomando G., Gatti M., Caldarelli A., Lim D., Penengo L., Brunelleschi S. (2015). Nicotinamide phosphoribosyltransferase (NAMPT/PBEF/visfatin) is a tumoural cytokine released from melanoma. Pigment Cell Melanoma Res..

[164] Grozio A., Mills K.F., Yoshino J., Bruzzone S., Sociali G., Tokizane K., Lei H.C., Cunningham R., Sasaki Y., Migaud M.E. (2019). Slc12a8 is a nicotinamide mononucleotide transporter. Nat. Metab..

[165] Khaidizar F.D., Bessho Y., Nakahata Y. (2021). Nicotinamide Phosphoribosyltransferase as a Key Molecule of the Aging/Senescence Process. Int. J. Mol. Sci..

[166] Kim H.J., Kazi J.U., Lee Y.R., Nguyen D.H., Lee H.B., Shin J.H., Soh J.W., Kim E.K. (2010). Visualization of the melanosome transfer-inhibition in a mouse epidermal cell co-culture model. Int. J. Mol. Med..

[167] Kim B., Hwang J.S., Kim H.S. (2015). N-Nicotinoyl dopamine inhibits skin pigmentation by suppressing of melanosome transfer. Eur. J. Pharmacol..

[168] Allouche J., Rachmin I., Adhikari K., Pardo L.M., Lee J.H., McConnell A.M., Kato S., Fan S., Kawakami A., Suita Y. (2021). NNT mediates redox-dependent pigmentation via a UVB- and MITF-independent mechanism. Cell.

